# *N*′-Diphenyl­methyl­ene-2-hydroxy­benzohydrazide

**DOI:** 10.1107/S1600536809043530

**Published:** 2009-10-28

**Authors:** Ning-Ning Ji, Zhi-Qiang Shi

**Affiliations:** a Department of Chemistry, Taishan University, 271021 Taian, Shandong, People’s Republic of China; b Department of Materials Science and Chemical Engineering, Taishan University, 271021 Taian, Shandong, People’s Republic of China

## Abstract

The title compound, C_20_H_16_N_2_O_2_, was synthesized by the reaction of 2-hydroxy­benzohydrazide with diphenyl­methanone. The dihedral angle between the phenyl rings is 76.28 (11)°. The amino H atom is involved in an intra­molecular N—H⋯O hydrogen bond. In the crystal structure, the hydr­oxy groups and carbonyl O atoms form inter­molecular O—H⋯O hydrogen bonds, which link the mol­ecules into chains running along the *b* axis.

## Related literature

For general background to Schiff bases in coordination chemistry, see: Garnovskii *et al.* (1993 ▶); Musie *et al.* (2001 ▶); Paul *et al.* (2002 ▶); Anderson *et al.* (1997 ▶). For a related structure, see Ji & Shi (2008 ▶).
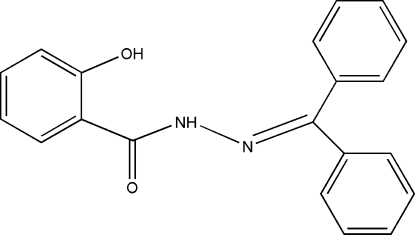


## Experimental

### 

#### Crystal data

C_20_H_16_N_2_O_2_
*M* *_r_* = 316.35
Monoclinic, 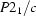

*a* = 15.4057 (18) Å
*b* = 12.5179 (15) Å
*c* = 8.8445 (10) Å
β = 103.777 (2)°
*V* = 1656.6 (3) Å^3^
*Z* = 4
Mo *K*α radiation
μ = 0.08 mm^−1^
*T* = 295 K
0.15 × 0.12 × 0.10 mm

#### Data collection

Bruker APEXII CCD area-detector diffractometer
Absorption correction: multi-scan (*SADABS*; Bruker, 2005 ▶) *T*_min_ = 0.988, *T*_max_ = 0.992
8538 measured reflections
2934 independent reflections
1910 reflections with *I* > 2σ(*I*)
*R*_int_ = 0.034

#### Refinement

*R*[*F* ^2^ > 2σ(*F* ^2^)] = 0.044
*wR*(*F* ^2^) = 0.126
*S* = 1.05
2934 reflections
219 parameters
H-atom parameters constrained
Δρ_max_ = 0.15 e Å^−3^
Δρ_min_ = −0.15 e Å^−3^

### 

Data collection: *APEX2* (Bruker, 2005 ▶); cell refinement: *SAINT* (Bruker, 2005 ▶); data reduction: *SAINT*; program(s) used to solve structure: *SHELXTL* (Sheldrick, 2008 ▶); program(s) used to refine structure: *SHELXTL*; molecular graphics: *SHELXTL*; software used to prepare material for publication: *SHELXTL*.

## Supplementary Material

Crystal structure: contains datablocks global, I. DOI: 10.1107/S1600536809043530/cv2634sup1.cif

Structure factors: contains datablocks I. DOI: 10.1107/S1600536809043530/cv2634Isup2.hkl

Additional supplementary materials:  crystallographic information; 3D view; checkCIF report

## Figures and Tables

**Table 1:** Hydrogen-bond geometry (Å, °)

*D*—H⋯*A*	*D*—H	H⋯*A*	*D*⋯*A*	*D*—H⋯*A*
O1—H1⋯O2^i^	0.82	1.90	2.7204 (17)	173
N1—H1*A*⋯O1	0.86	2.08	2.696 (2)	128

Symmetry code: (i) 
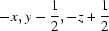
.

**Fig. 1.**
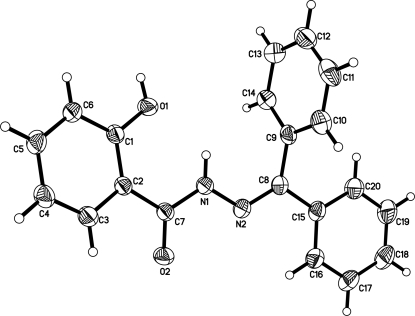
The molecular structure of (I), with displacement ellipsoids drawn at the 50% probability level.

**Fig. 2.**
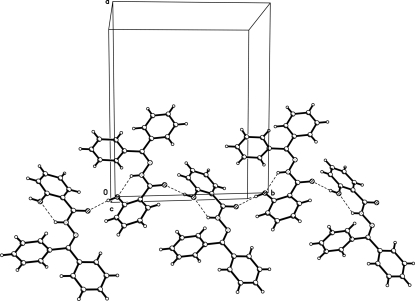
A portion of the crystal packing showing the hydrogen-bonded (dashed lines) chain.

## supplementary crystallographic information

### Comment

In recent years, a number of Schiff-bases have been investigated in terms of their coordination chemistry (Garnovskii *et al.*, 1993; Musie *et al.*, 2001; Paul *et al.*, 2002) and biological systems (Anderson *et al.*, 1997). In order to search for new Schiff-bases with higher bioactivity, the title compound, (I), was synthesized and its crystal structure determined.

In (I) (Fig. 1), the bond lengths and angles are in a good agreement with those observed in the related compound (Ji & Shi, 2008). Intramolecular O—H···N hydrogen bond (Table 1) influences the molecular conformation. In the crystal structure, the molecules are linked into infinite chains by O—H···O hydrogen bonds (Table 1, Fig. 2).

### Experimental

The title compound was synthesized by the reaction of 2-hydroxy-benzoic acid hydrazide(1 mmol, 152.2 mg) with diphenyl-methanone (1 mmol, 182.2 mg) in ethanol (20 ml) under reflux conditions (348 K) for 5 h. The solvent was removed and the solid product recrystallized from tetrahydrofuran. After six days, colourless crystals suitable for X-ray diffraction study were obtained.

### Refinement

All H atoms were placed in idealized positions (C—H = 0.93— 0.97 Å, N—H = 0.86 Å, O—H = 0.82 Å) and refined as riding atoms, with *U*_iso_(H) = 1.2 or 1.5*U*_eq_ of the parent atom.

### Crystal data

**Table:**

C_20_H_16_N_2_O_2_	*F*(000) = 664
*M**_r_* = 316.35	*D*_x_ = 1.268 Mg m^−^^3^
Monoclinic, *P*2_1_/*c*	Mo *K*α radiation, λ = 0.71073 Å
Hall symbol: -P 2ybc	Cell parameters from 1519 reflections
*a* = 15.4057 (18) Å	θ = 2.7–21.7°
*b* = 12.5179 (15) Å	µ = 0.08 mm^−^^1^
*c* = 8.8445 (10) Å	*T* = 295 K
β = 103.777 (2)°	Block, colourless
*V* = 1656.6 (3) Å^3^	0.15 × 0.12 × 0.10 mm
*Z* = 4	

### Data collection

**Table:**

Bruker APEXII CCD area-detector diffractometer	2934 independent reflections
Radiation source: fine-focus sealed tube	1910 reflections with *I* > 2σ(*I*)
graphite	*R*_int_ = 0.034
φ and ω scans	θ_max_ = 25.1°, θ_min_ = 1.4°
Absorption correction: multi-scan (*SADABS*; Bruker, 2005)	*h* = −18→16
*T*_min_ = 0.988, *T*_max_ = 0.992	*k* = −14→13
8538 measured reflections	*l* = −10→10

### Refinement

**Table:**

Refinement on *F*^2^	Secondary atom site location: difference Fourier map
Least-squares matrix: full	Hydrogen site location: inferred from neighbouring sites
*R*[*F*^2^ > 2σ(*F*^2^)] = 0.044	H-atom parameters constrained
*wR*(*F*^2^) = 0.126	*w* = 1/[σ^2^(*F*_o_^2^) + (0.0607*P*)^2^ + 0.0637*P*] where *P* = (*F*_o_^2^ + 2*F*_c_^2^)/3
*S* = 1.05	(Δ/σ)_max_ < 0.001
2934 reflections	Δρ_max_ = 0.15 e Å^−^^3^
219 parameters	Δρ_min_ = −0.15 e Å^−^^3^
0 restraints	Extinction correction: *SHELXTL* (Sheldrick, 2008), Fc^*^=kFc[1+0.001xFc^2^λ^3^/sin(2θ)]^-1/4^
Primary atom site location: structure-invariant direct methods	Extinction coefficient: 0.0070 (15)

### Special details

**Table:**

Geometry. All e.s.d.'s (except the e.s.d. in the dihedral angle between two l.s. planes) are estimated using the full covariance matrix. The cell e.s.d.'s are taken into account individually in the estimation of e.s.d.'s in distances, angles and torsion angles; correlations between e.s.d.'s in cell parameters are only used when they are defined by crystal symmetry. An approximate (isotropic) treatment of cell e.s.d.'s is used for estimating e.s.d.'s involving l.s. planes.
Refinement. Refinement of *F*^2^ against ALL reflections. The weighted *R*-factor *wR* and goodness of fit *S* are based on *F*^2^, conventional *R*-factors *R* are based on *F*, with *F* set to zero for negative *F*^2^. The threshold expression of *F*^2^ > σ(*F*^2^) is used only for calculating *R*-factors(gt) *etc*. and is not relevant to the choice of reflections for refinement. *R*-factors based on *F*^2^ are statistically about twice as large as those based on *F*, and *R*- factors based on ALL data will be even larger.

### Fractional atomic coordinates and isotropic or equivalent isotropic displacement parameters (Å^2^)

**Table:**

	*x*	*y*	*z*	*U*_iso_*/*U*_eq_	
O1	0.00834 (9)	0.02500 (10)	0.30160 (15)	0.0574 (4)	
H1	−0.0088	−0.0359	0.3125	0.086*	
O2	0.06286 (9)	0.32928 (10)	0.16995 (15)	0.0555 (4)	
N1	0.12496 (10)	0.18665 (12)	0.30963 (18)	0.0502 (4)	
H1A	0.1222	0.1197	0.3297	0.060*	
N2	0.19612 (10)	0.24715 (12)	0.38673 (19)	0.0528 (4)	
C8	0.26326 (12)	0.19876 (15)	0.4739 (2)	0.0482 (5)	
C2	−0.01536 (12)	0.16611 (13)	0.1159 (2)	0.0422 (4)	
C7	0.05922 (12)	0.23391 (14)	0.2017 (2)	0.0434 (5)	
C9	0.27034 (12)	0.08062 (15)	0.4971 (2)	0.0484 (5)	
C6	−0.10938 (12)	0.00942 (15)	0.0696 (2)	0.0502 (5)	
H6	−0.1254	−0.0570	0.1016	0.060*	
C1	−0.03865 (12)	0.06532 (14)	0.1631 (2)	0.0431 (5)	
C15	0.34002 (12)	0.26687 (16)	0.5508 (2)	0.0520 (5)	
C3	−0.06406 (13)	0.20669 (15)	−0.0252 (2)	0.0521 (5)	
H3	−0.0496	0.2738	−0.0570	0.063*	
C16	0.34477 (13)	0.37350 (16)	0.5095 (2)	0.0586 (6)	
H16	0.2984	0.4033	0.4343	0.070*	
C5	−0.15573 (13)	0.05148 (16)	−0.0693 (2)	0.0577 (5)	
H5	−0.2029	0.0133	−0.1309	0.069*	
C17	0.41724 (15)	0.43560 (19)	0.5786 (3)	0.0684 (6)	
H17	0.4193	0.5068	0.5499	0.082*	
C20	0.41002 (13)	0.22566 (19)	0.6647 (3)	0.0659 (6)	
H20	0.4081	0.1548	0.6951	0.079*	
C4	−0.13280 (14)	0.15061 (16)	−0.1188 (2)	0.0601 (6)	
H4	−0.1636	0.1785	−0.2139	0.072*	
C10	0.32717 (14)	0.02240 (18)	0.4281 (2)	0.0635 (6)	
H10	0.3609	0.0570	0.3685	0.076*	
C14	0.22226 (14)	0.02769 (16)	0.5863 (3)	0.0620 (6)	
H14	0.1841	0.0658	0.6336	0.074*	
C12	0.28481 (17)	−0.13853 (18)	0.5365 (3)	0.0733 (7)	
H12	0.2891	−0.2123	0.5489	0.088*	
C13	0.23000 (16)	−0.08149 (18)	0.6065 (3)	0.0746 (7)	
H13	0.1977	−0.1163	0.6682	0.089*	
C18	0.48636 (16)	0.3929 (2)	0.6896 (3)	0.0767 (7)	
H18	0.5356	0.4347	0.7348	0.092*	
C11	0.33363 (16)	−0.08721 (19)	0.4479 (3)	0.0739 (7)	
H11	0.3713	−0.1262	0.4008	0.089*	
C19	0.48253 (15)	0.2882 (2)	0.7335 (3)	0.0791 (7)	
H19	0.5288	0.2593	0.8098	0.095*	

### Atomic displacement parameters (Å^2^)

**Table:**

	*U*^11^	*U*^22^	*U*^33^	*U*^12^	*U*^13^	*U*^23^
O1	0.0607 (9)	0.0447 (8)	0.0559 (9)	−0.0078 (7)	−0.0078 (7)	0.0078 (7)
O2	0.0594 (9)	0.0384 (7)	0.0623 (9)	−0.0028 (6)	0.0021 (7)	0.0022 (6)
N1	0.0429 (9)	0.0383 (8)	0.0611 (10)	−0.0037 (7)	−0.0043 (8)	0.0002 (8)
N2	0.0446 (9)	0.0491 (9)	0.0586 (10)	−0.0067 (8)	0.0000 (8)	−0.0011 (8)
C8	0.0404 (11)	0.0523 (11)	0.0488 (11)	−0.0012 (9)	0.0042 (9)	0.0004 (9)
C2	0.0399 (10)	0.0395 (10)	0.0448 (11)	0.0032 (8)	0.0056 (8)	−0.0019 (8)
C7	0.0413 (10)	0.0396 (11)	0.0472 (11)	0.0028 (9)	0.0059 (9)	−0.0006 (9)
C9	0.0382 (10)	0.0516 (11)	0.0494 (12)	0.0018 (9)	−0.0011 (9)	−0.0013 (9)
C6	0.0461 (11)	0.0439 (11)	0.0562 (12)	−0.0032 (9)	0.0038 (10)	−0.0039 (9)
C1	0.0422 (10)	0.0405 (10)	0.0433 (11)	0.0032 (8)	0.0034 (9)	−0.0025 (8)
C15	0.0426 (11)	0.0608 (13)	0.0500 (12)	−0.0057 (10)	0.0055 (9)	−0.0017 (10)
C3	0.0526 (12)	0.0480 (11)	0.0514 (12)	0.0015 (9)	0.0038 (10)	0.0028 (9)
C16	0.0492 (12)	0.0623 (13)	0.0614 (14)	−0.0066 (11)	0.0078 (10)	0.0012 (11)
C5	0.0510 (12)	0.0580 (13)	0.0564 (13)	−0.0049 (10)	−0.0025 (10)	−0.0116 (11)
C17	0.0616 (14)	0.0704 (14)	0.0731 (16)	−0.0205 (12)	0.0156 (13)	−0.0029 (12)
C20	0.0542 (13)	0.0691 (14)	0.0652 (14)	−0.0085 (11)	−0.0041 (11)	0.0014 (11)
C4	0.0604 (13)	0.0600 (13)	0.0493 (12)	0.0007 (11)	−0.0078 (10)	0.0012 (10)
C10	0.0580 (14)	0.0745 (15)	0.0561 (13)	0.0080 (12)	0.0096 (11)	−0.0011 (12)
C14	0.0535 (13)	0.0533 (12)	0.0791 (15)	0.0034 (10)	0.0157 (11)	0.0031 (11)
C12	0.0687 (16)	0.0562 (14)	0.0826 (17)	0.0063 (12)	−0.0065 (14)	−0.0007 (13)
C13	0.0647 (15)	0.0598 (14)	0.0981 (19)	−0.0009 (12)	0.0170 (14)	0.0087 (13)
C18	0.0557 (15)	0.0946 (19)	0.0743 (16)	−0.0265 (14)	0.0043 (13)	−0.0112 (14)
C11	0.0684 (16)	0.0753 (16)	0.0708 (16)	0.0239 (13)	0.0021 (13)	−0.0180 (13)
C19	0.0538 (14)	0.0948 (19)	0.0756 (17)	−0.0127 (13)	−0.0108 (12)	−0.0011 (14)

### Geometric parameters (Å, °)

**Table:**

O1—C1	1.363 (2)	C16—C17	1.378 (3)
O1—H1	0.8200	C16—H16	0.9300
O2—C7	1.231 (2)	C5—C4	1.389 (3)
N1—C7	1.352 (2)	C5—H5	0.9300
N1—N2	1.373 (2)	C17—C18	1.373 (3)
N1—H1A	0.8600	C17—H17	0.9300
N2—C8	1.285 (2)	C20—C19	1.381 (3)
C8—C15	1.485 (3)	C20—H20	0.9300
C8—C9	1.493 (3)	C4—H4	0.9300
C2—C3	1.390 (3)	C10—C11	1.384 (3)
C2—C1	1.402 (2)	C10—H10	0.9300
C2—C7	1.483 (2)	C14—C13	1.380 (3)
C9—C14	1.374 (3)	C14—H14	0.9300
C9—C10	1.388 (3)	C12—C13	1.362 (3)
C6—C5	1.371 (3)	C12—C11	1.369 (3)
C6—C1	1.390 (3)	C12—H12	0.9300
C6—H6	0.9300	C13—H13	0.9300
C15—C20	1.388 (3)	C18—C19	1.372 (3)
C15—C16	1.390 (3)	C18—H18	0.9300
C3—C4	1.372 (3)	C11—H11	0.9300
C3—H3	0.9300	C19—H19	0.9300
			
C1—O1—H1	109.5	C6—C5—H5	119.7
C7—N1—N2	119.00 (15)	C4—C5—H5	119.7
C7—N1—H1A	120.5	C18—C17—C16	120.4 (2)
N2—N1—H1A	120.5	C18—C17—H17	119.8
C8—N2—N1	118.12 (16)	C16—C17—H17	119.8
N2—C8—C15	116.33 (17)	C19—C20—C15	121.1 (2)
N2—C8—C9	124.85 (17)	C19—C20—H20	119.5
C15—C8—C9	118.78 (16)	C15—C20—H20	119.5
C3—C2—C1	118.30 (17)	C3—C4—C5	118.98 (19)
C3—C2—C7	115.88 (16)	C3—C4—H4	120.5
C1—C2—C7	125.80 (16)	C5—C4—H4	120.5
O2—C7—N1	121.23 (17)	C11—C10—C9	119.9 (2)
O2—C7—C2	120.69 (16)	C11—C10—H10	120.0
N1—C7—C2	118.00 (16)	C9—C10—H10	120.0
C14—C9—C10	118.84 (19)	C9—C14—C13	120.7 (2)
C14—C9—C8	121.65 (17)	C9—C14—H14	119.7
C10—C9—C8	119.51 (18)	C13—C14—H14	119.7
C5—C6—C1	120.47 (18)	C13—C12—C11	119.9 (2)
C5—C6—H6	119.8	C13—C12—H12	120.0
C1—C6—H6	119.8	C11—C12—H12	120.0
O1—C1—C6	121.44 (16)	C12—C13—C14	120.3 (2)
O1—C1—C2	118.79 (16)	C12—C13—H13	119.9
C6—C1—C2	119.76 (17)	C14—C13—H13	119.9
C20—C15—C16	117.83 (19)	C19—C18—C17	119.7 (2)
C20—C15—C8	121.03 (18)	C19—C18—H18	120.1
C16—C15—C8	121.13 (18)	C17—C18—H18	120.1
C4—C3—C2	121.93 (18)	C12—C11—C10	120.4 (2)
C4—C3—H3	119.0	C12—C11—H11	119.8
C2—C3—H3	119.0	C10—C11—H11	119.8
C17—C16—C15	120.9 (2)	C18—C19—C20	120.1 (2)
C17—C16—H16	119.6	C18—C19—H19	120.0
C15—C16—H16	119.6	C20—C19—H19	120.0
C6—C5—C4	120.55 (19)		
			
C7—N1—N2—C8	−171.19 (17)	C9—C8—C15—C16	167.06 (18)
N1—N2—C8—C15	177.98 (15)	C1—C2—C3—C4	−0.7 (3)
N1—N2—C8—C9	0.5 (3)	C7—C2—C3—C4	177.72 (17)
N2—N1—C7—O2	1.2 (3)	C20—C15—C16—C17	0.7 (3)
N2—N1—C7—C2	177.88 (15)	C8—C15—C16—C17	−178.39 (18)
C3—C2—C7—O2	17.5 (3)	C1—C6—C5—C4	−0.1 (3)
C1—C2—C7—O2	−164.16 (17)	C15—C16—C17—C18	0.2 (3)
C3—C2—C7—N1	−159.20 (16)	C16—C15—C20—C19	−0.7 (3)
C1—C2—C7—N1	19.1 (3)	C8—C15—C20—C19	178.36 (19)
N2—C8—C9—C14	−73.8 (3)	C2—C3—C4—C5	1.6 (3)
C15—C8—C9—C14	108.8 (2)	C6—C5—C4—C3	−1.2 (3)
N2—C8—C9—C10	106.6 (2)	C14—C9—C10—C11	0.9 (3)
C15—C8—C9—C10	−70.8 (2)	C8—C9—C10—C11	−179.48 (19)
C5—C6—C1—O1	180.00 (17)	C10—C9—C14—C13	−0.3 (3)
C5—C6—C1—C2	0.9 (3)	C8—C9—C14—C13	−179.9 (2)
C3—C2—C1—O1	−179.66 (16)	C11—C12—C13—C14	1.1 (4)
C7—C2—C1—O1	2.1 (3)	C9—C14—C13—C12	−0.8 (4)
C3—C2—C1—C6	−0.5 (3)	C16—C17—C18—C19	−1.0 (4)
C7—C2—C1—C6	−178.80 (16)	C13—C12—C11—C10	−0.5 (4)
N2—C8—C15—C20	170.39 (19)	C9—C10—C11—C12	−0.6 (3)
C9—C8—C15—C20	−12.0 (3)	C17—C18—C19—C20	1.0 (4)
N2—C8—C15—C16	−10.6 (3)	C15—C20—C19—C18	−0.1 (4)

### Hydrogen-bond geometry (Å, °)

**Table:**

*D*—H···*A*	*D*—H	H···*A*	*D*···*A*	*D*—H···*A*
O1—H1···O2^i^	0.82	1.90	2.7204 (17)	173
N1—H1A···O1	0.86	2.08	2.696 (2)	128

Symmetry codes: (i) −*x*, *y*−1/2, −*z*+1/2.
